# Associations among Metabolism, Circadian Rhythm and Age-Associated Diseases

**DOI:** 10.14336/AD.2016.1101

**Published:** 2017-05-02

**Authors:** Yiwei Cao, Rui-Hong Wang

**Affiliations:** Faculty of Health Science, University of Macau, Macau, China; Faculty of Health Science, University of Macau, Macau, China

**Keywords:** Age-associated diseases, circadian rhythm, metabolic reprogramming, sirtuin, tumorigenesis, Warburg effect

## Abstract

Accumulating epidemiological studies have implicated a strong link between age associated metabolic diseases and cancer, though direct and irrefutable evidence is missing. In this review, we discuss the connection between Warburg effects and tumorigenesis, as well as adaptive responses to environment such as circadian rhythms on molecular pathways involved in metabolism. We also review the central role of the sirtuin family of proteins in physiological modulation of cellular processes and age-associated metabolic diseases. We also provide a macroscopic view of how the circadian rhythm affects metabolism and may be involved in cell metabolism reprogramming and cancer pathogenesis. The aberrations in metabolism and the circadian system may lead to age-associated diseases directly or through intermediates. These intermediates may be either mutated or reprogrammed, thus becoming responsible for chromatin modification and oncogene transcription. Integration of circadian rhythm and metabolic reprogramming in the holistic understanding of metabolic diseases and cancer may provide additional insights into human diseases.

## 1. Introduction

Modern society faces age associated health care problems globally. Age-associated abnormality in metabolic processes and functions lead to propensity to cancer and an array of metabolic diseases posing a huge challenge to human health. Metabolic diseases include obesity, diabetes and cardiovascular disease. In recent years, epidemiological studies have suggested a surprising association between metabolic diseases and an increased risk of multiple types of cancer, and have recommended precautionary measures [1-3] (Table 1). For example, a molecular mechanism of tumorigenesis in obesity has been proposed, involving secretion of adipokines (leptin, adiponectin and increased inflammatory cytokines) and secondary effects of obesity that increase anabolic pathways, such as insulin-insulin growth factor (IGF) signaling [4]. However, the biological mechanisms remain poorly understood, which hinders the development of therapeutic strategies for cancer prevention and treatment in patients with metabolic diseases.

At the cellular level, metabolism provides essential energy and substrates for maintenance of cellular function, support of cell growth and stimulation of cell proliferation. Reprogramming of glucose metabolism, represented by enhanced glycolysis and glucose uptake, has been considered as a hallmark of cancer in recent years [5]. Further, the abnormal metabolism in cancer cell may have a crucial role in the discovery of new therapeutic targets. In addition to the tendency of cancer cells to accumulate glutamine, acetate has also been highlighted as a new carbon source for consumption during cancer cell metabolism [6]. The disproportionate usage of these fuel sources illustrates a metabolic adaptation to allow tumor proliferation and is a consequence of metabolic reprogramming. The hypotheses proposed to decipher this reprogramming include oncogenes driving the aberration of specialized metabolic pathways, metabolic enzyme mutations and epigenetic modifications.

In biological systems, in addition to metabolism, circadian rhythms can also adapt to environmental change, and display conservative modulation in response to day/night, feed/starvation, warm/cold, and so on [7]. The latest report indicates that longer exposure to light fluctuates circadian rhythms and induces detrimental effects on biological processes[8]. And even use of electronic devices that emit light, could be enough to affect the circadian clock [9]. An intertwinement between circadian rhythms and metabolic diseases has also been proposed [10]. The emerging evidence indicates that the intrinsic clock is tightly coupled with different aspects of metabolic processes [11]. Endocrine factors that mediate metabolism are also orchestrated by the circadian clock, disorders of which influence physiological parameters such as blood pressure, as well as endocrine secretion, cell cycle, DNA repair etc. [12]. Based on this knowledge, chronotherapy has been applied for treatment of various diseases, for example in anticancer treatment, leading to optimized therapy plans for cancer patients [13]. The complex interactions between circadian rhythms, metabolism and human diseases are expected to shed light on the study of tumorigenesis and aging, which may have association with behavior, nutrient supplementation and energy consumption.

**Table 1:** The recent epidemiological studies relating metabolic disease and cancer risk.

Metabolic disease	Cancer risk	Country/Population	Reference
Hyperglycemia	Renal cell and liver cancer in men; Endometrial and pancreatic cancers in women are increased	Europe; Taiwan	[161, 162]
Diabetes	Colon, liver, pancreatic, endometrial and kidney, esophagus, rectum (F), stomach (F), thyroid (F), brain (F), lung (F), bladder, biliary tract and ovary cancer risks are increased	Sweden; Netherlands; Australia; African Americans; Native Hawaiians, Japanese Americans; Taiwan, Italy; USA; Japan; Hong Kong; Netherlands; Canada	[161-175]
Metabolic syndrome	Colorectal neoplasm; Prostate cancer; liver cancer risks are increased	Taiwan; Canada; England	[177-179]
Obesity	Esophageal, thyroid, liver, biliary tract, colorectal, ovary, gastric, breast, prostate, lung cancer risks are increased	USA; Europe; Japan; African American; Australia; Italy	[180-185]
Metformin use	Reduced breast, prostate, colorectal cancer risk and mortality, increased survival with gastric cancer	Canada; USA; Denmark; Taiwan; Korea	[186-191]

## 2. Initialization of Warburg effect

Almost a hundred years ago, Otto Warburg demonstrated how cancer cells predominately consume glucose through aerobic glycolysis, generating lactic acid even in the presence of oxygen [14]. The process observed in cancer cells result in glycolytic rates up to two hundred-fold higher than that in normal cells. Warburg postulated that dysfunctional mitochondria were the main cause of this change, and has been later called Warburg effect [15]. This interpretation was widely accepted by Warburg’s contemporaries and evolved in this century into a theory of adaptation to hypoxic conditions in pre-malignant lesions [16]. However, the root of this typical cancer metabolism has been examined further by scientists recently and the complexity underlying the mechanism of the Warburg effect is being unraveled [17, 18].

It appears that cell reprogramming may take place in cancer cells, and is responsible for metabolic change. Mounting evidence shows that oncogene/tumor suppressors are involved in this process [19, 20]. For example, the oncogene Myc and hypoxia-inducible factor 1 (HIF-1) have been reported to regulate most of the glycolytic enzymes that lead to enhanced glycolysis. In addition to the secondary effects on cancer cells, driven by metabolic reprogramming, enhanced anabolic metabolism would also appear in environments rich in nutrients and growth factors [21, 22]. More promisingly, in recent years, metabolic enzymes and metabolites have been reported to be involved in regulating gene transcription in addition to their roles in metabolism, for example oncoprotein (PKM2) and oncometabolite (2HG). This control of oncogene and tumor suppressor transcription by metabolic enzymes will be discussed in following sections.

## 3. Cell proliferation and metabolism are tightly constrained by each other

Using unbiased screening for cDNA that can immortalize mouse embryonic fibroblasts (MEFs), scientists have identified two kinds of glycolytic enzymes, phosphoglycerate mutase (PGM) and glucosephosphate isomerase (GPI), which can enhance glycolysis, allowing uncontrolled proliferation and escaping Ras-induced arrest [23]. This observation led to a hypothesis that glycolytic activation contributes to immortalization of cells. Following this, another group of scientists published a study in *Science* that demonstrated a 4- to 5-hour metabolic cycle (alternating between glycolysis and respiration) in yeast cultured in a nutrient-limited medium, which mimics growth conditions in the wild. DNA replication occurred only in the glycolytic phase [24]. Moreover, disruption of DNA checkpoint kinase, which links the cell cycle to the circadian rhythm, will lead to synchrony between the metabolic cycle and the cell division cycle. This also shed light on the associations among metabolism, the circadian rhythm and cell proliferation. The circadian clock has been proposed as a bridge between metabolism and cancer [25].

**Figure 1.**
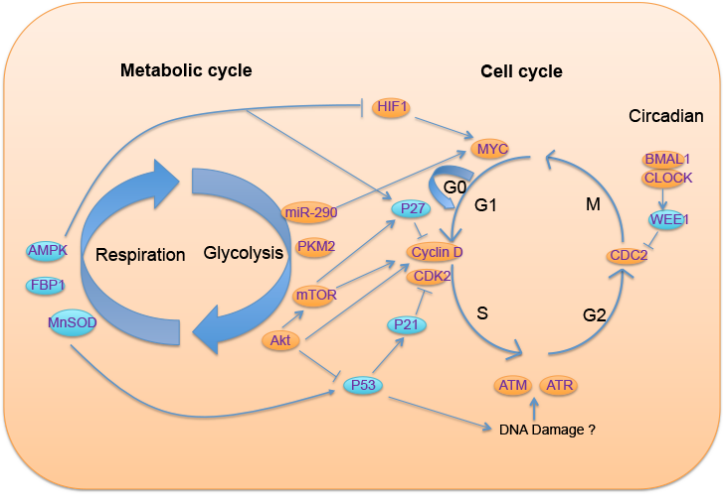
The metabolic cycle gates cell cycle entry On the left is the metabolic cycle with two phases, glycolysis and respiration; different colors of circular icon represent either suppressors (blue) or oncogenes in tumorigenesis (orange); on the right is the cell cycle, in which the regulatory relationship of mediators between these two cycles have been illustrated.

That cell proliferation and metabolism are tightly constrained with each other give us a clue about the mediators involved in this situation. This point can be illustrated with the following examples: when a cell is proliferating, a mediator is needed to “tell” the metabolism that glycolysis is an appropriate method to choose for now. In contrast, when the cell begins glycolysis to prepare the substrates for macromolecular synthesis, a signal can be transferred to the cell cycle with “ready” messages. Therefore, it is not difficult to understand that oncogenes and tumor suppressors can regulate metabolic pathways. For instance, a key function of Myc in tumor cells is to promote utilization of glutamine in order to provide an extra nitrogen and carbon source to fulfill rapid proliferation of the cells, and the function of tumor suppressor p53 is mediated by nutrient deprivation that increases the expression of p53 isoform and relocalization of region-binding protein 1 (SMAR) [26]. In contrast, the metabolic enzymes Akt and AMP-activated protein kinase (AMPK) are now considered as oncogene and tumor suppressor, respectively. Furthermore, reports have revealed common regulatory pathways that are shared between metabolism and proliferation in cancer cells [20]. Some groups targeting glutamine metabolism have demonstrated positive effects against tumor proliferation [27]. Therefore, question can be raised: can metabolic status gate cell cycle entry (Fig. 1)?

**Fig 2.**
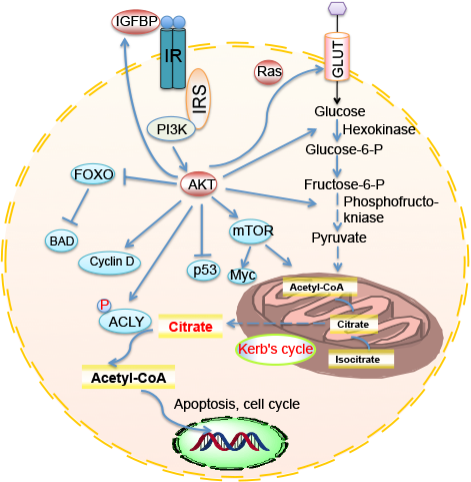
Enhanced anabolic PI3K/Akt pathway in a cancer cell PI3K/Akt/mTOR pathways are involved in 1. Glycolysis; 2. Cell growth; 3. Cell cycle; 4. Cell survival.

## 4. New advances in metabolism and cancer---glycolytic factors tuning tumorigenesis

The explanation of initialization of the Warburg effect can no longer be satisfied with the demands of rapid anabolic biosynthesis [18]. Evidence is mounting that metabolic pathways and signal transduction are reciprocally regulated, rather than as separate entities. This means not only that oncogenic signaling is regulated by increased nutrient uptake and disorders of cell metabolism, but also that the signal transduction pathway can be modified by metabolic status and has effects on cell physiology. This pathway of “metabolism gates cell cycle entry” involves: (a). the nutrient-sensor, AMP-activated protein kinase, AMPK [28] and anabolic pathways PI3K/Akt [29]; (b). Availability of “oncometabolites”, the metabolites involved in protein modification, such as acetyl-CoA, 2-hydroxyglutarate (2-HG) [30]; and (c). Glycolytic factors (enzymes, miRNA) that serve as mediators with multiple functions in both cell metabolism and tumorigenesis.

### 4.1 Anabolic pathways driving metabolic disorders in tumorigenesis

In a normal cell, growth, proliferation and differentiation are precisely controlled by a series of extra- and intracellular factors. Among them, growth factors directly signal cells to increase nutrient uptake and enhance anabolic metabolism [31]. Under the condition that growth factor signaling has been constitutively activated through an appropriate receptor, increased anabolic metabolism is observed in transformed cancer cells. Influenced by this, deregulation of several metabolic pathways has been proposed to be involved in tumorigenesis.

Many experiments have directly explored the effects of energy expenditure on tumor progression. Feeding *Drosophila* a high sugar diet led to enhanced tumor growth; a similar result was not found when the flies were fed a high-fat diet [32]. The tumorigenesis associated with a high-carbohydrate diet requires the insulin/PI3K pathway, which has been evaluated by both PI3K gene modification and pharmacologic approaches. Moreover, it has been found, via Wingless-mediated insulin receptor upregulation in a Ras/Src tumor model, that tissues are in fact insulin sensitive rather than resistant. This provides evidence that, although sugar might promote insulin resistance, it will also enhance tumor formation in an insulin/PI3K-dependent manner [32]. The activation of the PI3K/Akt/mTOR pathway has already been discussed as an oncogenic factor that has multiple effects, including provoking the Warburg effect (Fig. 2). Akt can phosphorylate and activate ATP-citrate lyase (ACLY), which is responsible for converting citrate back to acetyl-CoA in the cytoplasm. Owing to this process, it can prevent a cytosolic accumulation of citrate that negatively regulates glycolysis and maintains the intracellular level of acetyl-CoA. By inducing Gcn5p/SAGA-catalyzed histone acylation, which eventually influences transcription of growth factor, acetyl-CoA represents a critical metabolic intermediate and carbon source in biological processes that may also be involved in tumorigenesis [33]. Thus, cancer cells harbor an abnormal tendency to metabolize glutamine and acetate as carbon sources, which has been highlighted in recent years [6].

With regard to metabolic enzymes, studies have demonstrated the pivotal role of ACLY in cell proliferation and tumorigenesis *in vitro* and *in vivo* [34, 35]; it is also required for histone acylation in response to growth factor stimulation [36]. Higher expression of ACLY was indeed found in human lung adenocarcinoma [37]. Inhibition of ACLY resulted in cell growth arrest both in vitro and in vivo, showing its possible role in cancer therapy. Moreover, it has been found that, in addition to PI3K, another ligase (Skp2-SCF) can also regulate glycolysis and tumorigenesis through ubiquitination of Akt [38]. This testifies the core position of the Akt node in controlling the metabolic balance and normal physiology of cells by controlling a series of downstream signals, from cell growth (mTOR) and the cell cycle (cyclin D) to cell apoptosis (Bad) [3, 39]. In addition to ACLY, another Akt-activated glycolytic enzyme, hexokinase, responsible for phosphorylating glucose, has been demonstrated to be essential for nuclear repression of HXK1 (hexokinase) and GLK1 (glucokinase) in yeast and to modulate apoptosis through interaction with Bad [40, 41]. These are not the only examples of the multifaceted functions of a glycolytic enzyme [42], and more new evidence to testify that these glycolytic factors are involved in tumorigenesis remains to be explored.

Furthermore, it has been reported that Akt regulates the expression of insulin-like growth factor binding protein-3 (IGFBP-3) in human non-small cell lung cancer (NSCLC) cells [43]. IGFBP-3 induces G1 cell cycle arrest and apoptosis in several cancer cell lines, including human NSCLC [44]. The alteration of this protein by oncogenic Akt can be considered as a new cancer therapeutic target. Lastly, it has been reported that Akt activation is a key determinant of histone acetylation in tumor cells [45], which provides direct evidence of epigenetic alternation for a particular metabolic enzyme. This observation was also confirmed in human glioma and prostate cancer, in which AcH4 is associated with pAKT levels. In summary, enhanced anabolic effects centered on Akt appear to be a reliable pathway of abnormal metabolism caused by cancer.

**Figure 3.**
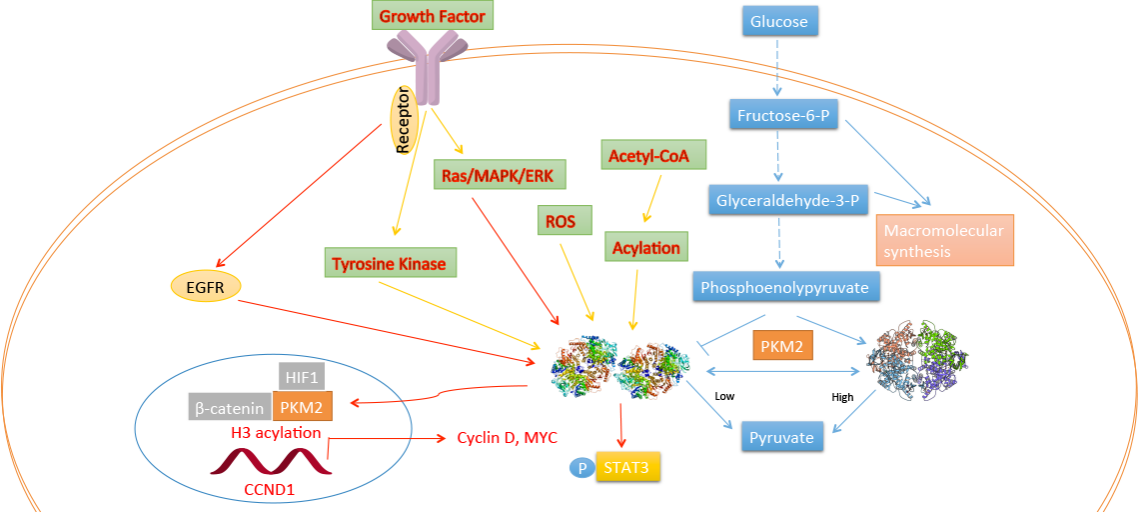
Regulation of PKM2 and its function in the nucleus.

AMPK, the nutrient/energy-sensor, plays a crucial role in various biological processes, and has been reviewed as a “metabolic checkpoint” [46]. It controls the ATP/AMP ratio and anabolic metabolism. Lack of AMPK promotes the Warburg effect and increases HIF-1a expression, which eventually causes lymphoma [47]. Because HIF-1 is downstream factor of mTORC1, it emphasizes the control of AMPK in oncogenic mTORC1 [48]. The most recent studies provide new insight that an alternative nitrogen source may regulate AMPK in controlling mTORC1 signaling, which might constitute a complement of AMPK/mTOR signaling in the metabolic response [49]. The yeast ortholog of AMPK has been shown to play a role in the regulation of global histone acylation and the amount of acetyl-CoA [50]. In mammals, AMPK activation is directly associated with H2B phosphorylation at serine 36 [51]. Recent findings suggest that AMPK directly phosphorylates O-linked β-*N*-acetylglucosamine (O-GlcNAc) transferase and suppresses histone H2B O-GlcNAcylation [52]. Importantly, AMPK can also directly phosphorylate p53 on serine and regulate the cyclin D inhibitor p27 by phosphorylation of Thr198, leading to indirect control of the cell cycle [53]. In addition, AMPK is also connected with cell autophagy and polarity, as reviewed recently [54]. Collectively, the role of AMPK as a tumor suppressor is well reported, and it cooperates with Akt, which can in turn phosphorylates and activates mTORC1, provides a representative anabolic pathway that is involved in cancer pathogenesis. Abnormal metabolic status may activate or inactivate one of these mediators linked with cellular metabolism and eventually lead to cancer.

### 4.2 Tumorigenesis through metabolic enzyme mutation

Through whole genome sequencing, scientists have identified mutations of isocitrate dehydrogenase 1 (IDH1, cytoplasm) and IDH2 (mitochondria) in gliomas and acute myeloid leukemia [55, 56]; the mutants have an enzymatic property of producing 2HG from alpha-ketoglutarate. The mutations are correlated with global histone methylation [57]; unexpectedly, 2HG metabolites inhibited histone demethylases, thus are considered to be oncometabolites [58]. Strikingly, pyruvate kinase (PK), the glycolytic enzyme that catalyzes transformation of phosphoenolpyruvate (PEP) to pyruvate, has been reported to be involved in tumorigenesis in its M2 isoform [59] (Fig. 3). In mammals, there are four isoforms of PK (M1, M2, L, and R) expressed in a tissue-specific manner; PKM2 is mainly expressed in fetal and cancer cells [60]. The PKM1 and PKM2 isoforms are produced by alternative splicing of the pre-messenger RNA on the *PKM2* gene [61]. When PKM2 is replaced with PKM1 in lung cancer cell lines by shRNA, the tumor formation in nude xenograft mice was reduced following decreased glycolytic metabolism, which confirmed the indispensable role of PKM2 in the Warburg effect and tumor growth [62]. This regulation could be a consequence of the function of PKM2 as a co-activator of HIF-1 [63]. As noted by Lu’s group, activation of epidermal growth factor receptor (EGFR) can induce the translocation of PKM2, wherein K433 of PKM2 binds to β-catenin at c-Src-phosphorylated Y333 and then assists recruitment at the CCND1 promoter and histone 3 acetylation, leading to cyclin D1 transcription. These researchers next revealed that this acetylation is required for PKM2 phosphorylation on histone H3 at threonine and subsequent expression of cyclin D1 and c-Myc, both of which are involved in cell proliferation and tumorigenesis as protein kinases [64-66]. This mechanism of EGFR induction of PKM2 may act through PKCε- and NF-κB-dependent activation [67]. Other evidence showed that PKM2 could phosphorylate mitosis checkpoint protein Bub3 at Y207, which is responsible for the spindle-assembly checkpoint and accurate chromosome segregation involved in EGF receptor activation during the induction of brain tumors [68].

Intriguingly, inactive PKM2 dimer can be regulated by transduction signaling, leading to Warburg effect and tumorigenesis, as demonstrated in recent years (Fig. 3). PKM2 has two forms, an active tetramer form (with high ligand affinity) and inactive dimer form which is prevented from binding to the cofactor fructose-1,6-bisphosphate (FBP) by tyrosine kinase signaling [59]. Hence, high glucose stimulates the acetylation of PKM2 on lysine 305, leading to a decrease in its active form. Meanwhile, its acetylation has been shown to be responsible for nuclear localization and tumorigenesis [69, 70]. Modification of PKM2 by phosphorylation and sumoylation can also lead to this translocation into the nucleus [66, 71]. Moreover, intracellular reactive oxygen species (ROS) can inhibit PKM2 by Cys^358^ oxidation which impairs the ontogenetic function of PKM2 [72]. The regulation by PKM2 enables the cell to adopt environmental changes, and its activation leads to nuclear protein kinase function.

**Table 2:** Non-metabolic functions of glycolytic factors

Name	Cancer	Metabolism	Modification	Reference
Lysine demethylase (LSD1)	Overexpressed in hepatocellular carcinoma	Glycolytic activity; Decreases mitochondrial metabolism genes	Methylate histone H3 at Lysine 4 in the promoter region	[192]
miR-122 microRNA	Breast cancer-secreted	Regulating the glycolytic enzyme PKM; Glucose uptake	Regulates glucose consumption in distant organs, including brain and lungs, and increases the incidence of metastasis	[92]
miR-290 miRNAs	Promotes pluripotency in PSCs	Up-regulates glycolytic enzymes Pkm2 and Ldha, stimulates glycolysis	miR-290 targets Mbd2, a reader for methylated CpGs, unregulated Myc	[193]
MnSOD-deficient mice	Skin carcinogenesis	Increased aerobic glycolysis	Increased uncoupling proteins (UCPs); p53	[194]
Fructose-1,6-bisphosphatase (FBP1)	FBP1 was suppressed in kidney tumours	FBP1 controls cell proliferation, glycolysis and the pentose phosphate pathway	Inhibits nuclear HIF function via direct interaction with the HIF inhibitory domain	[73]
6-phosphofructo-2-kinase/fructose-2,6-bisphosphatase 3 (pFKFB3)	Colon carcinoma	Glycolytic enzyme Induced by insulin	Regulates autophagy; increases cyclin-dependent kinase (Cdk)-1, Cdc25C, and cyclin D3; decreased the expression of the cell cycle inhibitor p27	[142] [195] [76] [77]
Type I transmembrane protein (MUC)	Pancreatic adenocarcinoma	Enhances glycolytic activity; enhances in vivo glucose uptake	MUC1 facilitates and stabilizes recruitment of HIF-1α and p300 on glycolytic gene promoters in a hypoxia-dependent manner	[74]
ENO1 (alpha-enolase)	Pancreatic cancer	Glycolytic enzyme	Alternative splicing form of ENO1, transcriptionally represses MYC	[75]

There is evidence suggesting that fructose-1, 6-bisphosphatase 1 (FBP1) enzyme antagonizes glycolytic flux and is usually decreased in cancer cells. Pan-metabolomic analysis has been used to show that the FBP1 protein was inhibited in both clear cell renal cell carcinoma (ccRCC) tumors and hepatocellular carcinomas; moreover, this disappearance was not regulated by HIF activation and exhibited the tumor-suppressive functions of FBP1. Finally, these researchers showed that HIF enzymatic activity could be directly inhibited by FBP1 through interaction with the HIF-α inhibitory domain [73]. These non-enzymatic functions of FBP1 implicate the metabolic regulation of hypoxic responses in tumor formation. In addition, a type I transmembrane protein, MUC1, was also reported to enhance glucose uptake and glycolysis by occupying a multiple gene promoter. It can also facilitate recruitment of HIF-1α on gene promoters which control glycolytic metabolism, thus being responsible for tumor metabolic changes [74]. Other glycolytic factors, summarized in Table 2, include the glycolytic enzyme ENO1 which has a similar gene sequence to Myc promoter-binding protein-1 (MBP-1) in chromosome 1 and eventually leads to transcriptional repression of the oncogene c-myc [75]. 6-Phosphofructo-2-kinase (PFKFB3), expression of which can be stimulated by insulin, is localized in the nucleus and controls expression of cell cycle proteins such as cyclin-dependent kinase (Cdk)-1, cyclin D3 and p27 [76, 77].

These discoveries provide crucial elements that show how the downstream cascade can interfere with upstream signaling homeostasis and provide a good example of how glycolytic factors are involved in gene transcription and tumor progression.

### 4.3 MicroRNAs serve as glycolytic regulators in tumorigenesis

MicroRNAs (miRNAs) are small non-coding RNA molecules, which are 17-24 nucleotides in length and which target sites on the 3′-untranslated regions of messenger RNA (mRNA) to provide post-transcriptional modification [77]. In recent years, they have been widely used as diagnostic markers for various cancers, including lung, ovarian and pancreatic cancers [78-81]. Their oncogenic and tumor suppressive roles have been described as intertwined with typical molecular pathways that are regulated by Myc, Ras and p53 [82]. Overexpression of miR-21 in cancer cells will up regulate HIF-1α and activation of the AKT and ERK pathways through targeting of PTEN [83]. In addition to miR-21, other microRNAs have roles in regulating HIF-1 and other oncogenes and tumor suppressors [84-87]. More interestingly, miRNAs that are reduced in tumors, when delivered by viral vectors, can serve as a therapeutic agent that suppresses tumorigenesis [88]. Among them, the microRNA let-7 tumor suppressor has surprisingly showed regulation of glucose metabolism through the Lin28/let-7 axis, targeting multiple components of the PI3K-mTOR pathway involving impaired glucose tolerance and insulin resistance [89]. Moreover, let-7a was found recently to be able to reprogram cancer metabolism by decreasing anabolic enzymes and upregulation oxidative phosphorylation genes [90]. In addition, miR-378 inhibited the expression of two PGC-1β partners, ERRγ and GABPA, leading to metabolic shift of cancer cells [91]. Most recently, miR-122 secreted by breast cancer cells was reported to be responsible for reprogramming glucose metabolism in remote organs in order to increase metastasis [92]. As circulating microRNAs are cosidered to be biomarkers for cancer prognosis and to modulate cell function, these characteristics identify them as emerging intercellular messengers and they may have a crucial role in tumorigenesis.

## 5. New advances in the circadian clock and metabolism

In addition to metabolism, another system can also sense the “outliers” of the environment: the circadian rhythm, which orchestrates the 24-hour day and night light cycle based on Earth’s gravitational field, has been proposed as a connection between metabolism and cancer [25]. However, its role in cancer cell transformation in response to metabolic cues has been underestimated for a long time. The circadian clock comprises a central clock, located in the hypothalamic suprachiasmatic nucleus (SCN), and peripheral clocks, both of which regulate various important biological processes. Importantly, circadian clock genes are expressed in almost all central nerve system (CNS) and peripheral tissues and may be responsible for autonomous cell alteration in different external environments. Because the circadian rhythm is intertwined with the metabolic rhythm, we hypothesize that disturbance of circadian rhythm will in turn activate cell-autonomous transformation to generate the Warburg effect. This could be significant because metabolic disease and cancer occur in multiple organs in the human body. More evidence about metabolism and the circadian rhythm in that has emerged in recent years is reviewed below.

### 5.1 Sirtuins at the crossroad that connects cancer, metabolism and circadian clock

Sirtuins are a NAD^+^-dependent protein family composed of seven protein members that occur in different compartments of living cells (SIRT3, SIRT4, SIRT5, located in mitochondria; SIRT1, SIRT6, SIRT7, in the nucleus; and SIRT2, mainly in the cytoplasm). Similar to their divergent locations, various enzymatic activities have also been demonstrated, ranging from lysine deacetylase and ADP-ribosyltransferase to deacylase[93]. SIRT1, the best understood sirtuin protein, requires NAD+ as a cofactor for its activity and has been noted in tumorigenesis, owing to its biological role in maintaining genome integrity and DNA repair. In murine models involving knockout or overexpression of different sirtuin genes, direct evidence has been found that defective sirtuin function may either promote or suppress cancer formation [94]. Although the precise role of sirtuin in tumorigenesis is not fully understood, it can be inferred that: a. as an advanced regulatory node in metabolism, cancer and aging, the maintenance of SIRT1 is fundamental in normal cell function; b. the deacetylation of histone and non-histone proteins by SIRT1 may also be an approach for cell reprogramming. Here, we demonstrate that the circadian clock can either work together with sirtuin to control chromatin remodeling, or affect their activity to contribute to disease pathogenesis.

**Figure 4.**
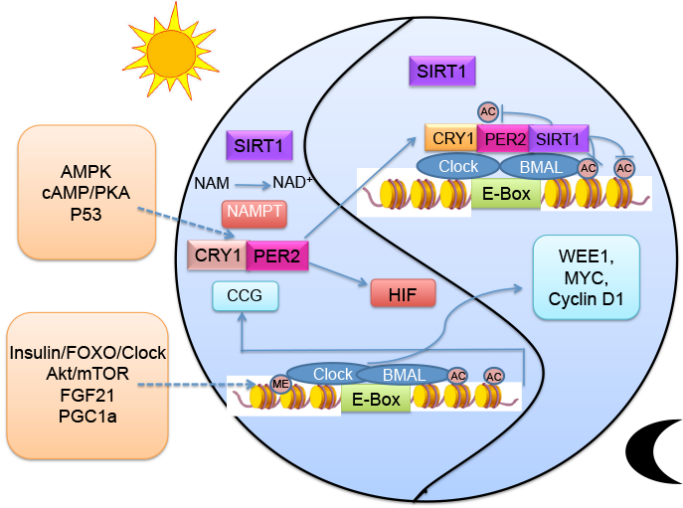
The transcriptional and post-translational loop of circadian systems

The circadian clock is controlled by a set of core clock genes. There are two heterodimeric transcription factors, CLOCK and BMAL1, that control rhythmic expression of many genes including its negative regulators, period (per1 and per2) and cryptochrome (cry1 and cry2). As shown by previous studies, the CLOCK itself has histone and BMAL1 acetyl transferase activity that is implicated in its regulatory functions in chromatin remodeling [95, 96]. Given that SIRT1 deacetylase activity is oscillating according to circadian rhythm, detailed research has demonstrated that SIRT1 directly interacts with CLOCK to control histone acetylation and regulate BMAL1 acetylation at Lys537 [97]. In addition, SIRT1 is also known to deacetylate PER2 and determine its stability. Supplementation with a series of SIRT1 activators, including SRT1720, has been found to repress clock genes both in vitro and in vivo and to have a chromatin modification effect which was SIRT1-dependent [98]. However, the activity of SIRT1 is dependent on its cosubstrate NAD^+^, which senses intracellular energy metabolism. NAMPT (nicotinamide phosphoribosyl-transferase), which catalyzes a rate-limiting step in the NAD^+^ salvage pathway, is regulated by CLOCK/BMAL1 transcription [99]. This pivotal finding has built a bridge between the classical transcription circadian loop and an enzymatic feedback loop, and has further demonstrated that metabolic disorder will interface with chromatin modification by the SIRT1-circadian system through the key metabolic regulator NAD^+^. Knockdown of CLOCK or BMAL1 induces hepatic insulin resistance, as demonstrated in a previous report. The authors found that CLOCK/BMAL1 are required for circadian expression of hepatic SIRT1, via binding to the SIRT1 promoter and controlling its expression [100]. This is how circadian oscillation influences metabolism through SIRT1 (Fig. 4). In contrast, environmental changes, including in metabolism, can also disrupt clock function through SIRT1. A report gives direct evidence that tobacco consumption leads to lung inflammation via SIRT1-dependent BMAL1 mediation, and this mutation was observed following chronic and acute exposure to tobacco in mice [101].

SIRT6 and SIRT3 have also been implicated in involvement with the circadian clock. The activity of SIRT3 in mitochondria is also mediated by the concentration of NAD^+^. As the circadian system involves rhythmic control of its rate-limiting enzyme NAMPT, as described above, it has also been found that the clock may regulate SIRT3 activity through the concentration of NAD^+^ and drive mitochondrial oxidative metabolism [102]. Once the circadian clock has been disrupted, the capacity for mitochondrial oxidative ability seems to be impaired, as detected by measurement of the oxygen consumption rate (OCR). The data indicate that Bmal1 mutants have defects in β-oxidation in addition to problems with pyruvate entry into the TCA cycle. This is a critical clue from which we can deduce the involvement of glycolysis and mitochondrial defects in cancer [103]. However, this is an indirect implication, and direct evidence needs to be obtained in future studies. Recent data also show that SIRT6 has different functions in regulating the CLOCK/BMAL1 network and also controls distinct hepatic circadian genes [104]. SIRT6 governs CLOCK/BMAL1 and recruitment of SREBP1 to circadian gene promoters and specifically regulates hepatic circadian transcription, which is correlated with downstream modulators of fatty acid metabolism [105].

Accumulating evidence has demonstrated that sirtuin may function either as oncogenes or tumor suppressors, influencing cell fate and metabolism during their modulation in cancer [106]. In this case, we provide details of interactions between sirtuin and the circadian clock via NAD^+^ concentration, demonstrating a more complicated situation in which sirtuin are involved in metabolic disorders and cancer. In addition, recent work has also found other rhythmic epigenetic modifications of the circadian clock, such as feedback actions of the PER complex on H3K9 di- and trimethylation [107]. In addition, histone methyltransferase mixed lineage leukemia 3 is controlled by the clock, which modulates over 100 epigenetic circadian outputs [108], demonstrating that the circadian clock alone is also important for sustaining temporal tissue physiology.

### 5.2 The circadian rhythm regulates metabolism and integrates nutrient signaling

Time-restricted feeding in mice will improve metabolic disease, even when they are fed a high-fat diet [109]. This provides a macroscopic view of how the circadian rhythm affects metabolism and may be involved in cell metabolism reprogramming and cancer pathogenesis. More evidence of how controlled feeding times influence the metabolism has been revealed [110]. Liver-specific deletion of Bmal1 in mice will lead to disrupted hepatic glucose regulatory genes and hypoglycemia [111]. Mice deficient in RORγ, another circadian gene, exhibit improvement of insulin sensitivity and glucose tolerance because of reduced hepatic gluconeogenesis [112]. This molecule can also occupy the citrate synthase promoter and regulate the expression of this key enzyme providing citrate derived acetyl-CoA [113]. This influence of lipogenesis is also indicated by another finding that binding of RORγ to ROREs controlled the transcription of lipid metabolic genes, such as insulin-induced gene 2a, Elovl3, *Insig2a* and sterol 12α-hydroxylase [114]. In clock mutant mice, the phenotype of impaired cholesterol metabolism is also observed and is involved in promotion of atherosclerosis [115]. Reviews have summarized the influence of the circadian clock on bile acid synthesis, lipogenesis, cardiovascular function, inflammation and, most importantly, glucose homeostasis [116]. Bioinformatics analysis has shown that most key rate-limiting enzymes involved in metabolism exhibit circadian rhythmicity, including those acting in glycolysis, gluconeogenesis, glycogenolysis, fatty acid synthesis, triglyceride storage, and cholesterol biosynthesis [117]. However, direct evidence of how the circadian systems regulate initialization of the Warburg effect is still lacking, although the clock indeed influences several curial metabolic enzymes and is an important factor in metabolic functions.

In turn, metabolic cues can also re-tune the performance of circadian oscillation. First, the nutrient sensor AMPK can phosphorylate the clock component cryptochrome 1 (CRY1), leading to its destabilization and rhythmic alteration [118]. In addition, casein kinases, also important modulators of circadian rhythms, can be phosphorylated by AMPK at Ser389 and thereby degrade PER2 [119]. This effect could also be observed after injection with metformin, but it could not be replicated in AMPK alpha2 knock-out mice. This regulatory function of AMPK in the circadian rhythm provides a striking example of nutrient signaling coupled with the human clock. Akt signaling was also found to alter the circadian rhythm in the SCN to lengthen rest-activity behavior [120]. Moreover, p53 binds directly to a response element in the PER2 promoter and hinders its expression. Mice deficient in p53 have a shorter, unstable period length under a light pulse [121]. The rhythmically expressed energy regulator PGC-1α stimulates the expression of Bmal1 and Rev-erbα through activation of orphan nuclear receptors and leads to a requirement for cell-autonomous clock reprogramming [122]. The direct connection between energy expenditure and the circadian cycle was demonstrated in 2013: feeding of a high-calorie diet influenced the circadian transcriptome and metabolome via impaired BMAL1 recruitment to its targeting chromatin sites and rhythmic recruitment of transcriptional factor PPARγ [123]. Through transcriptional remodeling, nutrients are directly linked to the circadian metabolome.

Post-transcriptional modification is also very important in circadian systems. Deacetylation-acetylations, described previously, are one possible functional biological modification that has the potential to influence the regulation of the circadian clock and is involved in metabolism. In one report, UBE3A disrupted oscillations by binding to BMAL1 and degrading it in a ubiquitin ligase-dependent manner [124]. In addition, the participation of histone methylation in the circadian clock has been investigated recently [108]. The occurrence of non-histone RNA methylation dependent on circadian control testified that m^6^A-RNA methylation sites are sufficient to generate a circadian rhythm that is sensitive to the amount of SAM (*S*-adenosyl-L-methionine) and SAH (*S*-adenosyl-L-homocysteine) [125]. This research was highlighted by several recent reviews [126, 127]. In an analogy with metabolic regulation, microRNA can also mediate circadian systems in response to environment alteration; for example, miR-219 was found to interact directly with the CLOCK-BMAL1 dimer, affecting the circadian pacemaker [128]. By facilitating phosphorylation in a GSK3β-dependent manner, the glucose sensor O-GlcNAcylation was shown to regulate clock and periodic protein transcriptional activities [129]. This promoter, being a nutrient sensor, shows how nutrients, especially glucose, fine-tune the circadian system and this provides strong evidence that transduction signaling is integrated with higher systems. Interestingly, fibroblast growth factor 21 (FGF21) and insulin also show the potential to the influence circadian rhythm [130-132]. The insulin-FOXO3-Clock signaling cascade has been described in recent years; it shows a mediating function in hepatic metabolism and oxidative sensitivity. This overlap of transduction signaling with metabolism provides a link by which outliers are integrated in both systems and play very important roles in the associations between metabolism, circadian rhythms and cancer.

### 5.3 Tissue-specific action of the circadian clock and its relation to cancer

Only 5%-10% of oscillatory transcripts are common between two given tissues (SCN, liver and muscle are involved), despite the fact that they share a highly conserved control system [133]. Using genetic techniques, scientists have observed cell type-specific clock gene knockdown phenotypes in 3T3-L1 adipocytes, MMH-D3 hepatocytes and other cell lines [134]. This involvement of CLOCK/BMAL1 may activate different sets of target genes in various tissues, as demonstrated in *Drosophila* [135]. Recent work done by Alexander’s group identified sequence motifs associated with CLOCK/BMAL1 binding sites unique to different parts of the body. One motif, the GATA factor binding site, has been revealed to be responsible for appropriate tissue-specific gene expression, thus providing a comprehensive picture of a clock gene selectively activating target gene transcription [136, 137]. More studies are necessary to identify binding sites of clocks in specific tissues, in order to analyze their effect on tissue-specific gene expression. Furthermore, tissue-specific actions have been observed in cell cycle control through cyclin-dependent kinase network, providing new insight into tumorigenesis in different organs [138]. The circadian influence on local physiology involves different types of epigenetic modification and may eventually cause different diseases, for example metabolic disease involving metabolic organs and cancers involving other organs. Like the example of clock protein DEC1 and DEC2, which is induced by CLOCK/BMAL1, control of metabolism and behavior by clock output is involved with hypoxia responses and carcinogenesis in a tissue-specific manner [139].

In the human population, CLOCK variants have been found to be associated with breast cancer risk, and CLOCK promoter hyper methylation will reduce breast cancer risk [140]. Clock is an independent risk factor for cancer; it is involved in control of cell proliferation, the DNA damage response, cellular senescence and the inflammatory response, in addition to metabolic homeostasis [141]. Overexpression of Bmal1 can increase the anticancer drug sensitivity of colorectal cancer [142]. Per2 mutation increased RAS-mediated oncogenic transformation and significantly changed the expression of the cell cycle genes p21 and cyclin D [66]. Per1 is being investigated in association with unfolded protein response (UPR), which has a role in tumorigenesis [143]. Interestingly, a recent report described that HCD feeding before tumor induction in mice was defected with ER-induced UPR [144], again showing the possible regulatory link between metabolism and the circadian clock in cancer onset. More specific molecular mechanisms for involvement of the clock in cancer require exploration, especially those acting with metabolism and the Warburg effect.

## 6. “Outliers” are also taken a role in aging process

Metabolic diseases and cancer are all age-associated diseases, with metabolism and circadian clock directly connecting with aging process. Decades ago, it was suggested that life-span was dependent on the rate of metabolic expenditure and oxidative damage [145]. Animals with calories restriction exhibit extension of average life span and lower oxidative damage rate. The balance between mitochondrial oxidant production and antioxidant can reflect mitochondrial respiration rate. Mitochondrial redox generation has long been implicated in the aging process [146]. Interestingly, treatment with metformin, a biguanide drug to treat type 2 diabetes, increases *C. elegans* life span by altering folate and methionine metabolism. Nevertheless, metformin had no effect on extending the life span of worms with 1% glucose supplement. The main target of metformin, AMPK, can activate SKN-1 that is essential in longevity [147]. As a regulator of metabolic homeostasis, sestrins are also described to have anti-aging function through AMPK-TORC1 axis [148]. These evidences imply that altered longevity are related to metabolic perturbations. Indeed, there is increased glycolysis in senescent fibroblasts, suggesting metabolic shift is also attributed to senescent cells and AMPK, which act as a sensor. AMPK can phosphorylate p53 or inhibits p21 or p16 transcription [149]. On the other hand, NAD^+^ and Sirt1 function together to regulate metabolism and circadian clock. Meanwhile, NAD^+^ level declined upon aging and restoring NAD^+^ could ameliorate many age-associated diseases[150]. Most recently, it has been found that NADase (CD38) expression was increased in multiple organs. CD38 deficient mice have higher level of NAD^+^ and are protected from aging related diseases. However, knockout of both CD38 and SIRT3 abolished the protective effects[151]. As NAD^+^ dependent deacetylases, increased sir2 is linked with extension of life span in worms; in mammals, deficiency of SIRT1 in MEF cells are resistant to senescence. Besides, Sirt6 deficiency will lead to age-related degeneration [152]. Accumulating evidence emphasized the central role of sirtuin between aging and metabolism.

**Figure 5.**
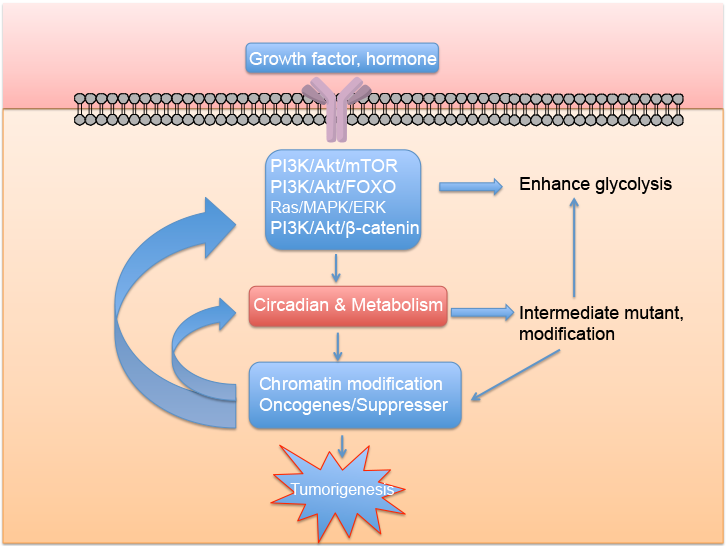
Proposed mechanism relating metabolic disease and cancer

Circadian clock has also been associated with control of aging. Disruption of circadian rhythm can accelerate aging. Resetting the clock accompanied with healthy diet increases longevity. Experimentally, deficient of BMAL1 will lead to premature aging and decreased life span because BMAL1 negatively control of mTORC1 signaling [153]. In the SCN, Sirt1 positively regulates genes controlling circadian clock and is reported to decline with aging [154]. Circadian gene per2 and sirt1 display a negative reciprocal relationship to regulate aging [155]. In sum, the interconnected clock and metabolism that incorporate transduction signaling, can in turn affect human aging process.

## 7. Prospective view

Accumulated evidence testifies that there are tight correlations between aging, cancer and metabolism [46, 156]. In this review, we have interpreted the association of these elements and included the concept of the circadian clock, stating that metabolism and the circadian rhythm may act together to contribute to tumorigenesis (Fig. 5). This review also provides some evidence that non-metabolic functions of glycolytic enzymes may be involved in tumorigenesis, and further research should be conducted in this area owing to its important roles in linking metabolism and cancer. Every metabolite is rhythmically circulated in human systems and their oscillation in both space and time may eventually destroys systemic homeostasis and causes physiological disorders. This effect occurs through multiple facets of interaction between the circadian clock and metabolism. Moreover, according to our findings in liver-specific SIRT1 knockout (Sirt1-LKO) mice, hepatic sirt1 deficiency will lead to hepatic glucose over-production, increasing circulatory glucose (hyperglycemia) and insulin resistance. Further understanding of the function of sirt1 in glucose metabolism has delineated a pathway in which sirt1 positively regulates transcription of Rictor and triggers phosphorylation of Akt at S473 and Foxo1 at S253. This is followed by negative regulation of the transcription of G6pase (glucose-6-phosphatase) and Pepck (phosphoenolpyruvate carboxykinase), which are responsible for gluconeogenesis [157]. Surprisingly, at a late stage in the study of sirt1 LKO mice aged over one year, lung tumors were observed by both gross observation and histological examination (unpublished data). It gives direct evidence between these two diseases. The circadian clock has a multilayer link with lung disease. It can regulate E-box mediated circadian activation of Nrf2 which uses antioxidant effects to modulate pulmonary fibrosis [158]. It regulates the inflammatory response in lung injury and also regulates glucocorticoid hormones involved in pulmonary inflammation [159, 160]. More evidence that metabolic disease leads to lung cancer is required; it may act through both metabolic reprogramming and disorder of the circadian system. From the review, we point out that all living organisms are immersed in more than one dimension in the universe. Consideration of both the quantity and the temporal challenge to the system might allow more precise deconstruction of a particular problem and leads to the truth, avoid practical mistakes. The integration of the circadian rhythm and metabolism with diseases is an example.
